# Simulating the Effects of Construction in NYC’s Chinatown on Fruit and Vegetable Consumption in Local Residents

**DOI:** 10.1093/geroni/igaa057.343

**Published:** 2020-12-16

**Authors:** Stella Yi, Yan Li, Valerie Imbruce, Yi-Ling Tan, Victoria Foster, Vivian Wang, Simona Kwon

**Affiliations:** 1 NYU School of Medicine, New York, New York, United States; 2 Icahn School of Medicine at Mount Sinai, New York, New York, United States; 3 Binghamton University, Binghamton, United States; 4 NYU School of Global Public Health, New York, New York, United States

## Abstract

In 2017, the mayor of New York City (NYC) unveiled a 10-year plan to close the city’s largest jail complex and to build four satellite detention centers – including one in Manhattan’s Chinatown. Chinatown is a destination for affordable produce and its retail produce sector is comprised of street vendors and small stores, a style of fresh fruit and vegetable (FV) marketing the city promotes to achieve its goal of equitable access to healthy foods. The objective of this study was to project the impact of the proposed construction activity on FV consumption among residents in Chinatown. We developed an agent-based model that accounts for individual and neighborhood-level factors (e.g., age, gender, education, food environment) to predict FV consumption at the neighborhood level in NYC. We assumed that long-term construction will lead to the closure/migration of fresh produce vendors and therefore a reduction of FV access. We simulated three scenarios in which the number of fresh produce vendors is reduced by 5%, 10%, and 15% due to construction. Results suggest that planned construction could decrease the consumption of FV by 2.1%, 4.4%, and 6.8% among residents in Chinatown if the construction would reduce the number of fresh produce vendors by 5%, 10%, and 15%, respectively. Preliminary sensitivity analyses demonstrate the negative impact of the construction on FV consumption could be greater among older (65+ years) vs. young adults. The planned construction of a detention center in Chinatown may decrease the consumption of FV among its residents, particularly older adults.

